# Tailoring Macro/Meso/Microporous Structures of Cellophane Noodle-Derived Activated Carbon for Electric Double-Layer Capacitors

**DOI:** 10.3390/ma17143474

**Published:** 2024-07-13

**Authors:** Hyeong-Rae Kim, Myeong-Hun Jo, Hyo-Jin Ahn

**Affiliations:** Department of Materials Science and Engineering, Seoul National University of Science and Technology, Seoul 01811, Republic of Korea; hyungrae_kim@seoultech.ac.kr (H.-R.K.); cmh6429@seoultech.ac.kr (M.-H.J.)

**Keywords:** cellophane noodle, activated carbon, KOH activation, porous structure, electric double-layer capacitor

## Abstract

To address the bottleneck associated with the slow ion transport kinetics observed in the porosity of activated carbons (ACs), hierarchically structured pore sizes were introduced on ACs used for electric double-layer capacitors (EDLCs) to promote ion transport kinetics under fast-rate charge–discharge conditions. In this study, we synthesized cellophane noodle-derived activated carbon (CNAC) with tailored porous structures, including the pore volume fraction of macro/meso/micropores and the specific surface area. The porous structures were effectively modulated by adjusting the KOH concentration during chemical activation. In addition, optimized KOH activation in CNAC modulated the chemical bonding ratios of C=O, pyrrolic-N, and graphitic-N. Given the hierarchically designed porous structure and chemical bonding states, the CNAC fabricated with optimized KOH activation exhibited a superior ultrafast rate capability in EDLCs (132.0 F/g at 10 A/g).

## 1. Introduction

With the rising demand for rechargeable energy storage cells, supercapacitors have received attention because of their high power density and specific capacitance [1]. Typically, supercapacitors consist of two facing electrodes, a separator, and an electrolyte. Supercapacitors are classified as electric double-layer capacitors (EDLCs), pseudocapacitors, and hybrid capacitors, depending on the type of electrode. Of these, EDLCs are the most commercialized supercapacitors because of their high power density, cyclic stability, and high productivity of active material [2]. EDLCs employ activated carbons (ACs) as active material because of their high specific surface area, pore volume, and electrochemical stability and low cost [3]. Nevertheless, novel approaches have been regularly attempted to develop high-performance ACs (particularly those with ultrafast rate capabilities) to meet social needs.

At the commercial level, ACs for supercapacitor applications are synthesized via the activation and carbonization of cokes or coconut shells. Recently, considerable research has focused on utilizing biomass waste as an AC precursor because of its cost-effectiveness and eco-friendliness [4]. Accordingly, the activation process has been tailored to the specific characteristics (carbon content, surface functionalities, nitrogen content, and skeleton structure) of biomass waste. Typically, pore formation on the AC surface occurs via a chemical or physical activation process [5]. Chemical activation involves the chemical reaction of ACs with oxidizing or dehydrating chemicals, such as KOH, NaOH, ZnCl_2_, H_3_PO_4_, and H_2_SO_4_ [6,7]. First, K, Na, or Zn oxides are formed on the surfaces of ACs through a wet process at elevated temperatures. The metal oxides are then removed from the carbon surface during carbonization, leaving porosity in the ACs. In the physical activation process, oxidizing gasses such as H_2_O, CO_2_, and O_2_ are used to create metal oxides on the AC surface [8]. Thus, the chemical activation process is feasible for use in controlling the surface functionalities, porous structures, and cost-effectiveness as compared with the physical activation process.

Based on the fact that charge storage in EDLC electrodes occurs on the AC surface according to physical adsorption/desorption, porosity plays a crucial role in transporting electrolyte ions at the interface between the AC and electrolyte [9]. Several studies have reported that the charge storage contribution varies depending on the pore size of the AC surface. Pores are generally classified into three types based on their diameters: macro (>50 nm), meso (2–50 nm), and micro (<2 nm) [10,11]. Macropores shorten the ion diffusion distance into the AC interior, mesopores promote fast ion transport through appropriately sized pores, and micropores expand the surface area for ion adsorption/desorption. However, to the best of our knowledge, few studies have reported the correlation between ion transport kinetics and the porous structure, including pore size distribution.

In this study, we synthesized cellophane noodle-derived activated carbons (CNACs) with tailored porosities and chemical bonding states for the first time. The cost-effectiveness of cellophane noodles (~USD 7 per 1 kg) and the scalability of the KOH activation process prompted us to use cellophane noodles as a raw material for AC and KOH as a chemical activation precursor. The pore volume fractions of the macro/meso/micropores were precisely controlled by adjusting the KOH concentration during chemical activation. Accordingly, we proposed an optimized porous structure for use in ultrafast EDLC electrodes. In addition, optimized KOH activation in cellophane noodle-derived carbon altered the chemical bonding states with enhanced ratios of C=O, pyrrolic-N, and graphitic-N. Consequently, the unique porous structure and chemical bonding state could significantly improve the performance of EDLC electrodes, particularly their ultrafast rate capabilities.

## 2. Materials and Methods

First, cellophane noodles (Ottogi Co., Ltd., Seoul, Republic of Korea) were heated at 400 °C for 2 h in a box furnace. Next, the cellophane noodles were functionalized with nitric acid (HNO_3_, 70.0%, Junsei, Seoul, Republic of Korea) and then washed with deionized water. The surface-functionalized cellophane noodles were dried overnight at 80 °C. For the KOH activation process, KOH pellets (85.0%, Samchun, Seoul, Republic of Korea) were dissolved in deionized water at three concentrations, with the weight ratios of cellophane noodles and KOH being adjusted to 1:5, 1:6, and 1:7. Then, the same amount of dried cellophane noodle was mixed with the three KOH solutions at 120 °C for 24 h. After the mixing, the residues were dried overnight at 120 °C, resulting in a completely dried powder. The powder was next carbonized at 800 °C for 2 h in a nitrogen atmosphere. Finally, the three carbonized powders were washed with hydrochloric acid (HCl, 35.0%, Samchun) and deionized water to remove the residual reactants. The activated carbon powders were then dried at 80 °C for 24 h, resulting in CNAC with three KOH activation concentrations of 1:5, 1:6, and 1:7 (denoted as CNAC15, CNAC16, and CNAC17, respectively). For comparison, cellophane noodle-derived carbon (CNC) was prepared and subjected to carbonization without KOH activation.

The morphologies of the fabricated CNC, CNAC15, CNAC16, and CNAC17 samples were observed using high-resolution scanning electron microscopy (HR-SEM, SU8010, Tokyo, Japan). Thermogravimetric analysis (TGA, DTG-60H, Greifensee, Switzerland) and derivative thermos gravimetry (DTA, DTG-60H, Greifensee, Switzerland) were conducted to examine the mass variation with increasing temperature in air. To demonstrate the porous structure, Brunauer–Emmett–Teller (BET, Belsorp-mini II, Osaka, Japan) and Barrett–Joyner–Halenda (BJH) analyses were conducted in a nitrogen atmosphere. The chemical bonding states and crystal structures were investigated using X-ray photoelectron spectroscopy (XPS, Cu Kα X-ray source, Multilab 2000, Lawrence, MA, USA), X-ray diffraction (XRD, Miniflex 300, Osaka, Japan) in a range of 10–80°, and Raman spectrometery (NRS-5100, Greifensee, Switzerland) with a laser-excitation wavelength of 532.1 nm.

To evaluate the electrochemical performances of the CNC, CNAC15, CNAC16, and CNAC17 samples, electrodes were fabricated by casting slurries on Ni foam (MTI Korea, Seoul, Republic of Korea). The composition of the slurries was adjusted to 70 wt% active material, 10 wt% conductive carbon (Ketjen black, Mitsubishi Chemical, Tokyo, Japan), and 20 wt% binder (polyvinylidene fluoride, Arkema, HSV900, Paris, France) in a N-methyl-2-pyrrolidinone solvent (Aldrich, St. Louis, MO, USA). After slurry casting, all the electrodes were dried at 80 °C for 8 h. The galvanostatic charge–discharge (GCD) process (WonATech, WMPG 1000S, Seoul, Republic of Korea) of the electrodes was conducted in the current density range of 0.2–10 A/g using a two-electrode measurement system with symmetric electrodes in a 6 M KOH solution as the electrolyte. Cyclic voltammetry (CV) curves were obtained in the potential range of 0–1.0 V, and electrochemical impedance spectroscopy (EIS) curves were obtained in the frequency range of 0.001–100 kHz using a potentiostat/galvanostat (Metrohm, Autolab PGSTAT302N, Greifensee, Switzerland).

## 3. Results and Discussion

**Figure 1:**
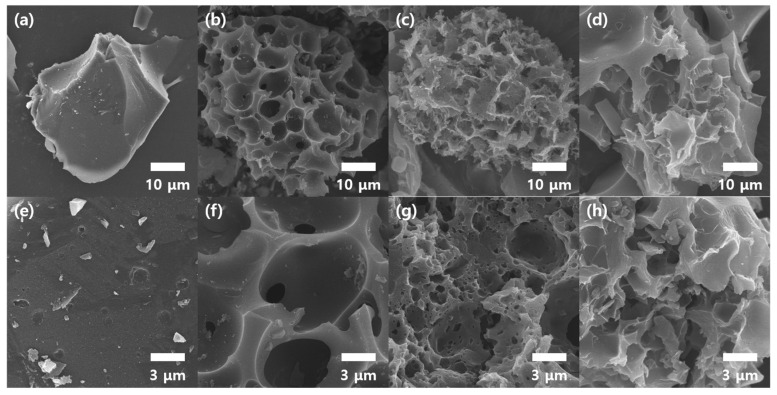
Low- and high-resolution SEM images: (**a**,**e**) CNC, (**b**,**f**) CNAC15, (**c**,**g**) CNAC16, and (**d**,**h**) CNAC17.

Electrode materials used in EDLC often encounter pore-associated bottlenecks, which suppress instantaneous ion transport during repetitive ultrafast charge–discharge cycles. To counteract this problem, we first introduced tailored porous structures ranging from macro-, meso-, and micro-scales into CNAC materials. The volume fraction of the macro/meso/micropores was effectively altered by adjusting the KOH concentration during the porosity-inducing activation. Figure 1 shows SEM images of CNC (Figure 1a,e), CNAC15 (Figure 1b,f), CNAC16 (Figure 1c,g), and CNAC17 (Figure 1d,h). As Figure 1a shows, CNC revealed a micron-sized particle (~38.6–43.3 μm) produced by grinding a mass of carbonized cellophane noodles. From the enlarged SEM image (Figure 1e), CNC revealed no specific porous structure on its surface due to the absence of the KOH activation process. By contrast, all CNAC samples showed porous morphologies produced by carbon decomposition reactions during KOH activation according to the following Equations (1)–(3) [12]:

6KOH + 2C → 2K_2_CO_3_ + 2K + 3H_2_ (1)

K_2_CO_3_ + C → K_2_O + CO_2_ (2)

2K + CO_2_ → K_2_O + CO (3)

K_2_O + C → 2K + CO (4)

Prior to KOH activation, the surface of the bare cellophane noodle was functionalized through a reaction with nitric acid. The surface functional groups on the cellophane noodle improved its dispersibility in water and promoted KOH activation, thus facilitating carbon decomposition. The functionalized cellophane noodle reacted with KOH at a lower temperature (120 °C), resulting in K_2_CO_3_, K, and H_2_ (Equation (1)). Following the reaction, the resulting K_2_CO_3_ and K were further decomposed into K_2_O during carbonization (800 °C) (Equations (2) and (3)). In addition, the K_2_O decomposed carbon into CO or CO_2_ (Equation (4)). Noticing that KOH is a key initiator in the robust activation of cellophane noodles, we controlled the KOH concentration to three levels during KOH activation. Remarkably, the three CNAC samples exhibited different porous morphologies. CNAC15 showed highly macroporous surface morphology (~0.9–8.7 μm), which was mainly attributed to the dominant reaction of Equation (1) (Figure 1b,f). The initial KOH reaction with carbon developed macro-scale porosity when the surface carbon of the cellophane noodle was removed. As further KOH activation proceeded during carbonization, the pore-forming reaction inside the cellophane noodle was accelerated through a series of carbon removal reactions (Equations (2)–(4)). Therefore, CNAC16 showed newly developed mesoporosity (~2–50 nm) in the macropores (Figure 1g). This mesoporous structure on the macropores provided ultrafast ion transport at the carbon surface by expanding low-resistance pathways for ion adsorption/desorption. However, CNAC17 exhibited a less porous morphology with decreased microporosity (Figure 1d,h). This may have been caused by the excessive amount of KOH, which inhibited the KOH activation reactions.

**Figure 2:**
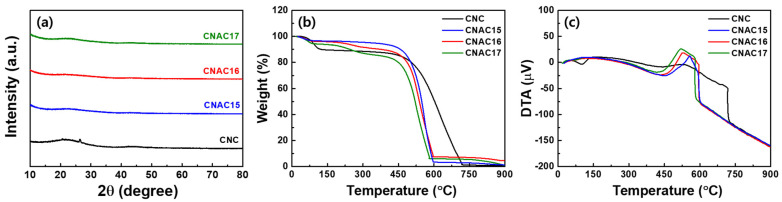
(**a**) XRD curves, (**b**) TGA curves, (**c**) DTG curves of CNC, CNAC15, CNAC16, and CNAC17.

As shown in the XRD results presented in Figure 2a, all samples exhibited a very broad peak near 25°, corresponding to the (002) plane of the carbon lattice. This broadening was slightly enhanced in the CNAC samples as compared with CNC, which was a result of KOH activation that degraded the sp^2^-hydridized bond on the carbon surface. The absence of specific peaks associated with potassium oxide confirmed that the resultant potassium oxides were completely removed during KOH activation. Figure 2b,c show TGA and DTG curves of all samples in a temperature range of 25–900 °C. In the TGA results, all samples exhibited sharp weight loss in the range of 450–750 °C, which resulted from the thermal pyrolysis of the carbon backbone and surface functional groups [13]. As the DTG curves (Figure 2c) show, all CNAC samples exhibited sharper exothermic peaks in the range of 450–600 °C as compared with the broad exothermic peak of CNC (450–750 °C). This acceleration of the exothermic peak in the CNAC samples was caused by the developed porous structure, which increased the pyrolysis rate of carbon at the surface as compared with that of CNC. Consequently, CNC reached a nearly zero (~0.7%) residual weight due to its high carbon purity. By contrast, the CNAC samples revealed specific residual weights following TGA (1.3%, 4.5%, and 1.0% for CNAC15, CNAC16, and CNAC17, respectively). The higher residual weight of CNAC16 could be attributed to the enhanced number of surface oxygen-containing functional groups resulting from the optimized KOH activation process.

**Figure 3:**
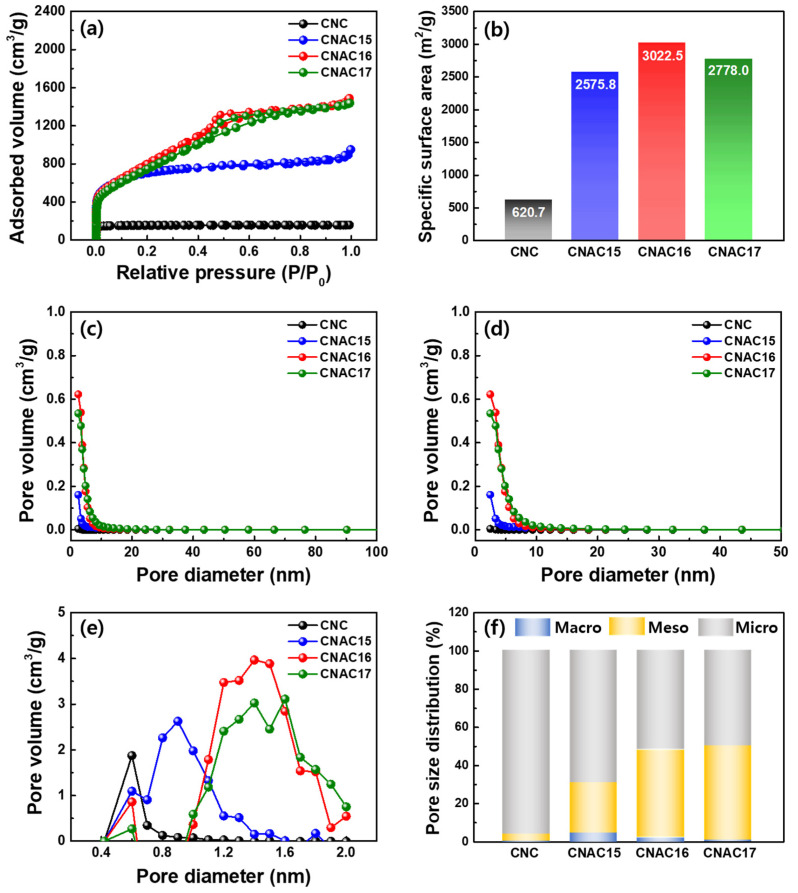
(**a**) N_2_ adsorption/desorption profile; (**b**) specific surface area values from BET results; (**c**) BJH plots in a pore diameter range of 2–100 nm; (**d**) BJH plots in a pore diameter range of 2–50 nm; (**e**) BJH plots in a pore diameter range of 0.4–2 nm; and (**f**) pore volume fractions according to the pore sizes of CNC, CNAC15, CNAC16, and CNAC17.

The porous structures and specific surface areas of all samples were demonstrated using BET and BJH analysis under the N_2_ adsorption/desorption process (Figure 3a–f). In general, the pores can be categorized into three types based on their diameter scale: macropores (>50 nm), mesopores (2–50 nm), and micropores (<2 nm). As Figure 3a shows, the CNC sample exhibited a low adsorption profile over the entire relative pressure range, which corresponded to the characteristic microporous structure according to the type-I isotherm of IUPAC [14]. By contrast, all CNAC samples exhibited a sharp increase in adsorption in the low P/P_0_ region (<0.05), indicating the presence of highly developed micropores on the surface. Significantly, CNAC16 and CNAC17 revealed a closed-loop profile with a gradually increasing adsorption volume in the middle P/P_0_ region (~0.1–0.8), which is characteristic of the mesoporous structure according to the type-IV isotherms. These adsorption/desorption profile results suggest that CNAC16 and CNAC17 had highly porous structures with enhanced mesoporosity as compared with CNC and CNAC15. From the BET results, specific surface area values of CNC, CNAC15, CNAC16, and CNAC17 were measured as 620.7, 2575.8, 3022.5, and 2778.0 m^2^/g, respectively (Figure 3b and Table 1). These specific surface areas demonstrate the optimized KOH activation effect of CNAC16, which accelerated the formation of mesopores and micropores through the increased KOH molecules for potassium oxidation, resulting in enhanced pore volumes. Figure 3c,d show the variations in the pore volume of all the samples according to their pore diameters. CNAC16 and CNAC17 exhibited higher pore volumes in the pore diameter range of 2–10 nm, demonstrating the development of mesoporous structures in CNAC16 and CNAC17 as compared with those in CNC and CNAC15. In addition, a closer investigation of the pore volume in the pore diameter range of 0.4–2 nm revealed higher pore volumes for CNAC16 and CNAC17 as compared with CNC and CNAC15. These results confirmed the highly developed microporous structures of CNAC16 and CNAC17 resulting from accelerated KOH activation. CNAC16 exhibited the highest total pore volume among all the samples (Table 1). To establish the porous structures of all samples, pore size distribution was calculated according to pore diameters based on the BET and BJH results (Figure 3f and Table 1). CNC showed a micropore-dominant structure (micropore volume fraction of 95.79%), which is not feasible in providing fast-rate ion transport. By contrast, CNAC15 exhibited an increased mesopore volume fraction (26.27%) due to the KOH activation effect. Notably, the mesopore volume fractions increased dramatically in CNAC16 (45.63%) and CNAC17 (49.02%) due to the accelerated KOH activation process, which formed large amounts of potassium oxides (K_2_CO_3_ and K_2_O) on the surface of the cellophane noodle. In general, potassium oxides are effectively pyrolyzed into CO and CO_2_ gasses during carbonization, leaving abundant meso-scale pores on the surface. It should be noted that the volume ratios of mesopores and micropores in CNAC16 and CNAC17 revealed different values, where CNAC16 exhibited a slightly higher micropore volume fraction and CNAC17 exhibited a similar volume ratio between the micropores and mesopores. This may have induced different ion adsorption/desorption transport kinetics at the surface. In particular, the synergies of abundant mesoporosity and a slightly higher micropore volume fraction are favorable to ultrafast charge transport kinetics and large amounts of charge adsorption/desorption, respectively.

**Figure 4:**
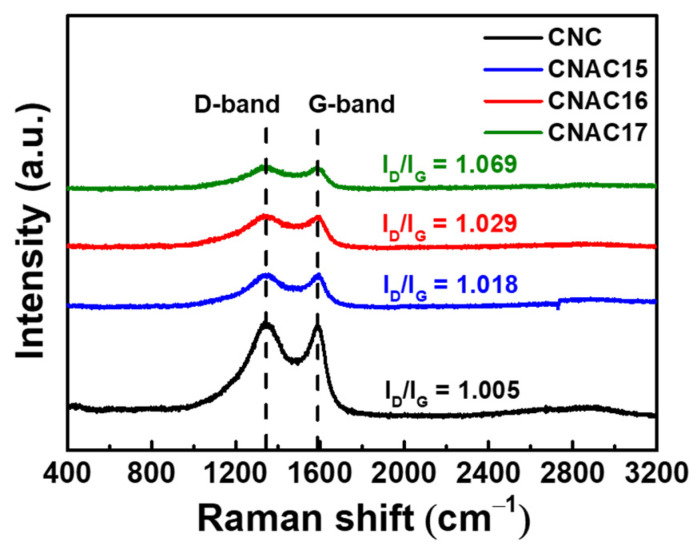
Raman spectra of CNC, CNAC15, CNAC16, and CNAC17 in a Raman shift range of 400–3200 cm^−1^, respectively.

Figure 4 exhibits Raman spectra of CNC and CNAC samples in a Raman shift range of 400–3200 cm^−1^. All samples presented common peaks at ~1343.9 and ~1589.1 cm^−1^, corresponding to the D-band and G-band, respectively [15]. Notably, CNC showed the lowest I_D_/I_G_ value (1.005) compared to those of CNAC samples. Considering that the D-band originates from defects in an sp^2^-hydridized carbon lattice, and the G-band results from a crystalline C-C bonded structure, the lowest I_D_/I_G_ value of CNC confirms the crystalline structure due to the absence of KOH activation process. In contrast, as the KOH concentration increased, the C-C decomposition on the surface of CNAC accelerated from CNAC15 to CNAC17, resulting in the increased I_D_/I_G_ values (1.018, 1.029, and 1.069 for CNAC15, CNAC16, and CNAC17, respectively). This result is consistent with the highly porous structure of CNAC samples compared to CNC due to the KOH activation, activating the carbon decomposition as shown in Equations (1)–(3).

**Figure 5:**
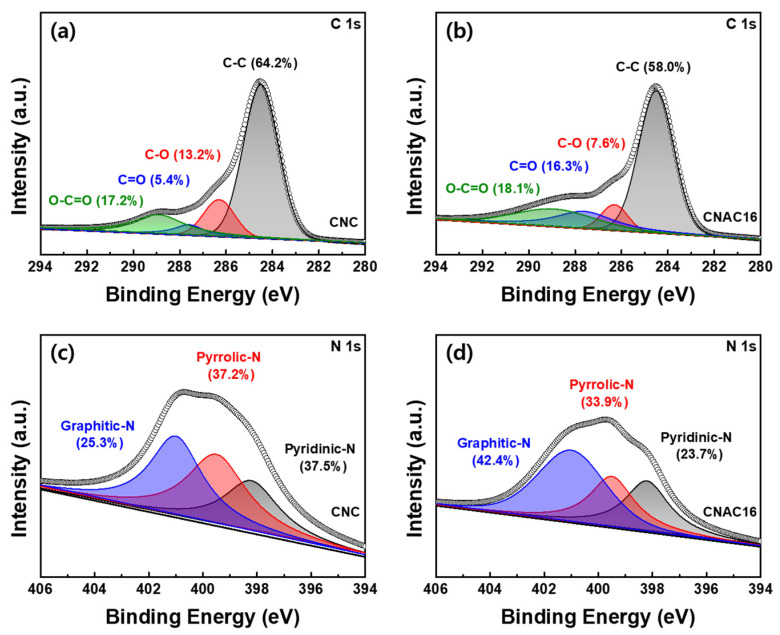
C 1s XPS spectra of (**a**) CNC and (**b**) CNAC16, and N 1s XPS spectra of (**c**) CNC and (**d**) CNAC16.

Figure 5a,b show the C 1s XPS profile of CNC and CNAC16, respectively. The C1s XPS spectra of the samples were deconvoluted to the chemical bonds C-C (~284.5 eV), C-O (~286.3 eV), C=O (~287.6 eV), and O-C=O (~288.9 eV) [16]. CNAC16 exhibited a significant decrease in C-C (58.0%) and a significant increase in C=O (16.3%) ratios as compared with those of CNC (64.2% and 5.4% for C-C and C=O, respectively). During KOH activation, an elevated amount of KOH attacked the carbonaceous surface, producing carbon vacancies. Next, −OH filled these carbon vacancies, generating C=O surface-functional groups on the CNAC16. This surface functionality, with enhanced surface C=O groups, lowered the resistance to ionic diffusion at the electrode/electrolyte interface due to the significant polarity of the C=O groups. Figure 5c,d show the N 1s XPS profiles of CNC and CNAC16, respectively. Both CNC and CNAC16 exhibited three deconvoluted bonding states: pyridinic-N (~398.2 eV), pyrrolic-N (~399.5 eV), and graphitic-N (~401.0 eV) [17]. The graphitic-N bonding ratio was noticeably higher for CNAC16 (42.4%) than for CNC (25.3%). Graphitic-N refers to the substitutional nitrogen atoms in the hexagonal carbon lattice, which donates additional electrons to the sp^2^-hybridized electron cloud. In general, this increase in electron density enhances the electrical conductivity of the active material, thereby improving the ultrafast rate capabilities of EDLC electrodes. In addition, the preservation of pyrrolic-N in CNAC16 (33.9%) as compared with that in CNC (37.2%) allowed electrolyte ions to adsorb/desorb on the surface through the significant polarity of pyrrolic-N groups. The enhanced chemical bonding states of the C=O groups, graphitic-N, and pyrrolic-N in CNAC16 could be expected to induce ultrafast charge transport kinetics in EDLC electrodes.

**Figure 6:**
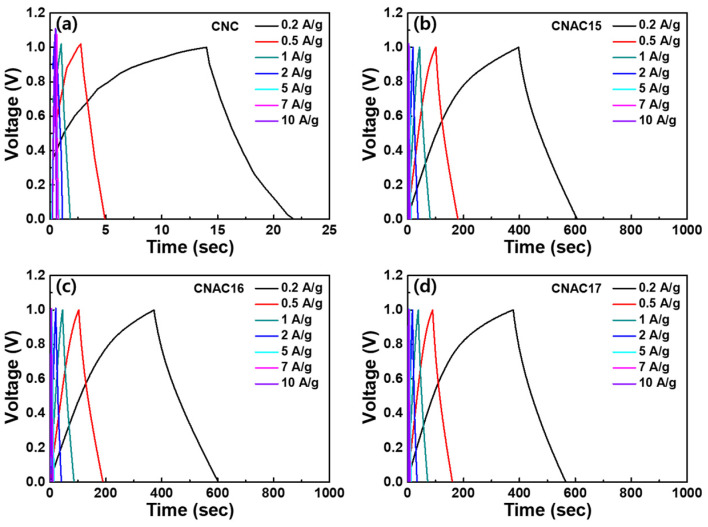
GCD curves at the applied current densities of (**a**) CNC and (**b**) CNAC15, (**c**) CNAC16, and (**d**) CNAC17 electrodes.

Figure 6 exhibits GCD curves of CNC and CNAC electrodes. All electrodes were fabricated by casting three slurries with the same composition, where the weight ratio of the active material, conductive carbon, and binder was 70:10:20. As a result, CNC showed noticeably shorter GCD times compared to those of CNAC. This is because the inferior porous structure of CNC reduced its electrochemical activity for electric double-layer formation during charge–discharge. Among the CNAC electrodes, CNAC16 exhibited the longest GCD times even under the high current densities, demonstrating the ultrafast rate capability of CNAC as the active material for ELDC. This result prompted us to calculate the specific capacitance for all electrodes.

**Figure 7:**
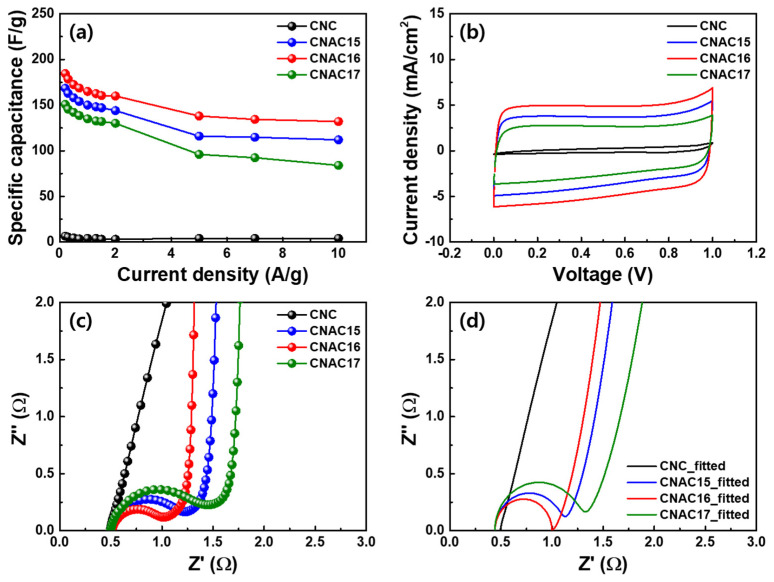
(**a**) Specific capacitance plots at the current densities of 0.2, 0.3, 0.5, 0.7, 1, 1.3, 1.5, and 2, 5, 7, and 10 A/g; (**b**) CV curves in the voltage range of 0–1 V at a scan rate of 100 mV/s; (**c**) EIS curves; and (**d**) fitted EIS curves of CNC, CNAC15, CNAC16, and CNAC17.

Figure 7a shows the specific capacitance plots according to the applied current densities of all electrodes. The specific capacitance was calculated from the resulting GCD curves in Figure 6 for all the electrodes using the following Equation (5) [18]:

Specific capacitance (F/g) = 4*I*/(*mdV*/*dt*) (5)

where *I*, *m*, *dV*, and *dt* are the current (A), weight of the active material (g), voltage window (V), and time required for discharge (s), respectively. CNC exhibited inferior specific capacitance values across the entire current density range due to the lack of porous structures on its surface (Table 2). By contrast, all the CNAC samples exhibited significantly increased specific capacitance values across the entire current density range. This increase in specific capacitance was due to the development of porous structures on their surfaces as a consequence of KOH activation. CNAC16 showed the highest specific capacitance values compared to those of CNAC15 and CNAC17, demonstrating its tailored porous structure and chemical bonding state for charge transport in the electrode. The increase in specific capacitance at a low current density (184.6 F/g at 0.2 A/g) was attributed to (i) an increased specific surface area due to improved micropore volume fraction and (ii) polar surface chemistry due to an enhanced C=O and pyrrolic-N bond ratio. These changes in CNAC16 accommodated large amounts of ion adsorption/desorption. The increase in specific capacitance at a high current density (132.0 F/g at 10 A/g) was attributed to (i) an enhanced mesopore volume fraction that promoted rapid ion diffusion at the carbon surface and (ii) an enhanced graphitic-N bond ratio that accelerated electron transfer in the carbon matrix. These changes in CNAC16 facilitated ultrafast charge transport at high current densities. Figure 7b shows the CV curves of all the electrodes in the voltage range of 0–1 V. As expected, the largest CV profile was observed for CNAC16 as compared with those of CNC, CNAC15, and CNAC17. This indicated that the highest electrochemical activity occurred with CNAC16, which was consistent with the specific capacitance results. In addition, among all the electrodes, the CV profile of CNAC16 exhibited a shape closest to a square, suggesting immediate charge transport in response to the applied voltage. Figure 7c shows the EIS curves of all the electrodes. In the figure, CNC shows no specific semi-circle, due to its inferior electrochemical activity, as confirmed by the specific capacitance results. By contrast, all CNAC electrodes show a semi-circle derived from charge adsorption/desorption at the surface of the active material. Remarkably, CNAC16 shows the smallest semi-circle, indicating the most resistively relaxed charge transport at the interface between the electrode and electrolyte [19,20,21]. Based on the EIS result, we fitted the EIS as shown in Figure 7d, and the resulting resistance values (series resistance, charge transfer resistance, and Warburg conductivity) are summarized in Table 3. As a result, all electrodes showed a similar level of series resistance due to the identical measurement system, including electrode and electrolyte. In particular, CNC showed a significantly lower charge transfer resistance (0.109 × 10^−3^ Ω) compared to those of CNAC electrodes, resulting from the inferior electrochemical activity at the electrode surface. Among the CNAC electrodes, CNAC16 exhibited the lowest charge transfer resistance (0.557 Ω), resulting from the enhanced surface functional groups (C=O and pyrrolic-N groups) and tailored porous structure (optimized pore volume ratio between the mesopore and micropore) compared to CNAC15 (0.644 Ω) and CNAC17 (0.825 Ω). The effective functional groups lowered the ion adsorption/desorption energy and the tailored porosity provided facile ion diffusion pathways at the CNAC/electrolyte interface. Therefore, CNAC16 revealed the highest Warburg conductivity (0.518 S) compared to CNC (4.6 × 10^−3^ S), CNAC15 (0.463 S), and CNAC17 (0.410 S). Tailored macro/meso/microporous structures and chemical bonding states were introduced into the CNAC by adjusting the KOH concentration during KOH activation. The tailored porous structures exhibited increased specific surface areas and pore volumes with enhanced mesopore and micropore volume fractions. This synergistic effect of mesopores and micropores not only accelerated ultrafast ion transport kinetics but also accommodated large amounts of charge adsorption/desorption in the EDLC electrodes. The surface chemistry rich in C=O and pyrrolic-N bonds promoted low-resistance ion diffusion due to their significant polarity. The graphitic-N doping configuration accelerated electron transfer under fast-rate charge–discharge conditions. Therefore, we believe that CNAC with tailored porous structures and chemical bonding states is a promising active material for ultrafast EDLC electrodes.

**Figure 8:**
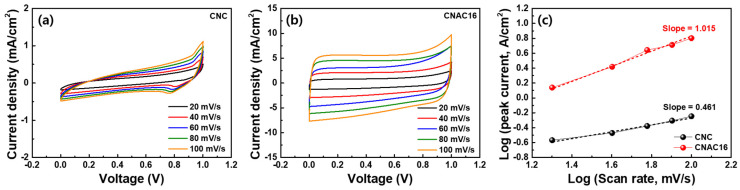
CV curves at the applied scan rate from 20 to 100 mV/s for (**a**) CNC and (**b**) CNAC16 electrode, respectively, and (**c**) log(*i*)–log(*v*) plots and slopes of CNC and CNAC16.

**Figure 9:**
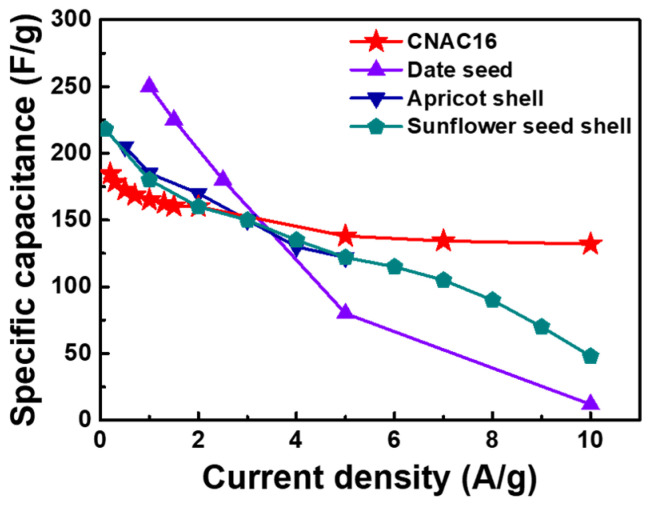
Comparison of specific capacitance plots of CNAC16 and previous biomass-derived activated carbon materials.

Figure 8a,b exhibit CV curves at the stepped scan rates from 20 to 100 mV/s for the CNC and CNAC16 electrode, respectively. CNAC16 showed a noticeably larger CV area and larger increase in current density for both the anodic and cathodic reaction compared to CNC. Moreover, the b-value was calculated using the following Equation (6) [22,23]:

*i* = a*v*^b^ (6)

where *i* is the peak current (A), *v* is the applied scan rate (V), and a and b are the adjustable constants. Based on the CV results, log(*i*)–log(*v*) values are plotted with the slopes of the fitted lines for CNC (0.461) and CNAC16 (1.015) (Figure 8c). Typically, b-values of 0.5 and 1.0 represent a process dominated by ion diffusion behaviors and capacitive-controlled behaviors of electrodes during the GCD process. Thus, the resulting b-values confirm the enhanced capacitive ion kinetics at the surface of CNAC16 compared to CNC. This supports the ultrafast rate capability of CNAC16 due to its tailored porous structures and chemical binding states. Moreover, the CNAC16 showed a superior ultrafast-rate capability compared to the previously reported biomass-derived activated carbon materials [24,25,26] (Figure 9).

**Figure 10:**
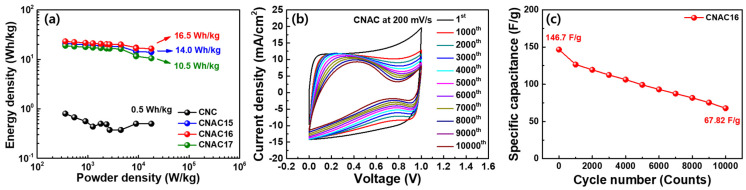
(**a**) Energy density plots of CNC and CNAC electrodes as a function of power density from 360 to 18,000 W/kg, (**b**) CV curves of CNAC16 during 10,000 consecutive cycles at 200 mV/s, and (**c**) specific capacitance plots calculated from the CV curves of CNAC16 during 10,000 cycles.

Figure 10a presents energy density plots according to the power densities for CNC and CNAC electrodes. The energy and power densities were calculated using the following Equations (7) and (8) [15]:

Energy density (Wh/kg) = Specific capacitance × (*dV*)^2^/8 (7)

Power density (W/kg) = Energy density/*dt* (8)

where *dV* and *dt* are the voltage gap (V) and discharge time (s), respectively. As a result, CNC exhibited very low levels of energy densities of 0.8 and 0.5 Wh/kg at the power densities of 360 and 18,000 W/kg, respectively. These energy densities imply an incompatibility of CNC as the active material for an EDLC electrode. In contrast, CNAC electrodes showed highly enhanced energy densities compared to CNC. Moreover, CNAC16 displayed high levels of energy densities of 23.1 and 16.5 Wh/kg at the power densities of 360 and 18,000 W/kg, respectively, compared to CNAC15 (21.0 and 14.0 Wh/kg at 360 and 18,000 W/kg, respectively) and CNAC17 (18.9 and 10.5 Wh/kg at 360 and 18,000 W/kg, respectively). This demonstrates the potential applicability of CNAC16 as an active material in high-performance EDLC. Figure 10b shows the CV curve changes in CNAC16 over 10,000 consecutive cycles at 200 mV/s. As a result, CNAC16 revealed a gradual decrease in CV area after the 10,000th cycle, which was due to the partial irreversibility of charge transport at the surface. This could have been influenced by the ultrafast scan rate condition (200 mV/s), which gradually degraded the ion diffusion kinetics at the surface. To elucidate the specific capacitance according to the long cycles, we calculated specific capacitance based on the CV curves during 10,000 cycles (Figure 10c). CNAC16 displayed a gradual decrease in specific capacitance from 146.7 to 67.82 F/g at the 1st and 10,000th cycle, respectively. Considering that the scan rate was very high, this slight degradation can validate the ultrafast longevity of CNAC16.

## 4. Conclusions

This is the first study on the fabrication of CNAC with tailored porous structures and chemical bonding states. During KOH activation, the porous structure and chemical bonding state were effectively altered by adjusting the KOH concentration to achieve a weight ratio of cellophane noodle to KOH of 1:6. The pore volume fraction of macro/meso/micropores (2.24%/45.63%/52.13%) was found to be an optimized porous structure with an enhanced specific surface area for high-performance EDLC electrodes. In particular, the synergistic effect of abundant mesoporosity and a slightly higher micropore volume fraction resulted in both ultrafast charge transport kinetics and large amounts of charge adsorption/desorption. In addition, the optimized KOH activation altered the chemical bonding states with enhanced ratios of C=O, pyrrolic-N, and graphitic-N bonds. The surface chemistry rich in C=O and pyrrolic-N bonds promoted low-resistance ion adsorption/desorption. The lattice chemistry rich in graphitic-N enhanced the fast electron transfer during ultrafast charge–discharge conditions. Given its unique porous structure and chemical bonding states, CNAC16 exhibited a superior ultrafast capability for use in EDLC electrodes.

## Figures and Tables

**Table 1:** BET and BJH results of CNC, CNAC15, CNAC16, and CNAC17.

Samples	S_BET_ (m^2^/g)	Total Pore Volume (cm^3^/g)	Pore Size Distribution		
			V_macro_ (%)	V_meso_ (%)	V_micro_ (%)
CNC	620.7	0.27	0.55	3.66	95.79
CNAC15	2575.8	1.63	4.92	26.27	68.81
CNAC16	3022.5	3.90	2.24	45.63	52.13
CNAC17	2778.0	3.64	1.05	49.02	49.93

**Table 2:** Specific capacitance results under different current densities of CNC, CNAC15, CNAC16, and CNAC17.

Samples	0.2 A/g	0.3 A/g	0.5 A/g	0.7 A/g	1 A/g	1.3 A/g	1.5 A/g	2 A/g	5 A/g	7 A/g	10 A/g
CNC	6.4	5.4	4.5	3.5	4.0	3.9	3.0	3.0	4.0	4.0	4.0
CNAC15	168.4	162.9	158.0	154.0	150.0	148.2	147.0	144.0	116.0	114.8	112.0
CNAC16	184.6	178.5	172.5	168.7	165.0	162.5	160.5	160.0	138.0	134.4	132.0
CNAC17	150.8	145.5	142.0	138.6	135.0	132.6	132.0	130.0	96.0	92.4	84.0

**Table 3:** Resistance values from EIS results of CNC, CNAC15, CNAC16, and CNAC17.

Samples	Series Resistance (Ω)	Charge Transfer Resistance (Ω)	Warburg Conductivity (S)
CNC	0.493	0.109 × 10^−3^	4.6 × 10^−3^
CNAC15	0.442	0.644	0.463
CNAC16	0.441	0.557	0.518
CNAC17	0.440	0.825	0.410

## Data Availability

The original contributions presented in the study are included in the article; further inquiries can be directed to the corresponding author.
